# Impedance Analysis of Chitin Nanofibers Integrated Bulk Acoustic Wave Humidity Sensor with Asymmetric Electrode Configuration

**DOI:** 10.3390/nano12173035

**Published:** 2022-09-01

**Authors:** Qiao Chen, Dong Liu, Xian-He Huang, Yao Yao, Kun-Lei Mao

**Affiliations:** School of Automation Engineering, University of Electronic Science and Technology of China, No. 2006, Xiyuan Avenue, Chengdu 611731, China

**Keywords:** humidity sensors, bulk acoustic wave sensor, chitin nanofiber (ChNF), mass sensitivity, fringing field effect, asymmetric electrode

## Abstract

This paper fabricated a high-performance chitin nanofibers (ChNFs)-integrated bulk acoustic wave (BAW) humidity sensor with an asymmetric electrode configuration. The ChNFs were successfully prepared from crab shells and used as moisture-sensitive materials to compare the performance of quartz crystal microbalance (QCM) humidity sensors with symmetric and asymmetric electrode structures. The QCM humidity sensor with a smaller electrode area exhibited high sensitivity of 58.84 Hz/%RH, competitive response/recovery time of 30/3.5 s, and low humidity hysteresis of 2.5% RH. However, it is necessary to choose a suitable electrode diameter to balance the stability and sensitivity because the impedance analysis result showed that the reduction of the electrode diameter leads to a sharp decrease in the Q value (stability). Next, the possible humidity-sensitive mechanism of the ChNFs-integrated asymmetric n-m electrode QCM humidity sensor was discussed in detail. Finally, the reasons for the highest sensitivity of the asymmetric n-m electrode QCM humidity sensors having a smaller electrode diameter were analyzed in detail in terms of both mass sensitivity and fringing field effect. This work not only demonstrates that the chitin nanofiber is an excellent potential material for moisture detection, but also provides a new perspective for designing high-performance QCM humidity sensors.

## 1. Introduction

The growing demand for precise humidity detection in many fields such as agricultural farming, medical monitoring, weather forecasting, and industrial manufacturing has contributed to the further development of humidity sensors [1,2,3]. Among the various humidity sensors currently available on the market and reported in the literature, the low-cost, miniaturized humidity sensors with real-time monitoring and digital transmission are particularly attractive [4,5,6,7]. In the past few decades, acoustic transducers including bulk acoustic wave (BAW) transducers, capacitive micromechanical ultrasonic transducers (CMUT), film bulk acoustic resonator (FBAR) transducers, and surface acoustic wave (SAW) transducers have been extensively developed in the field of gas and humidity sensing [8,9]. In particular, quartz crystal microbalance (QCM), as a typical BAW transducer, is a more desirable humidity transducer due to its ability to detect high sensitivity on the nanogram scale, simple structure, strong material selectivity, low cost, high interference immunity, and ability to withstand harsh environments [10,11].

**Figure 1:**
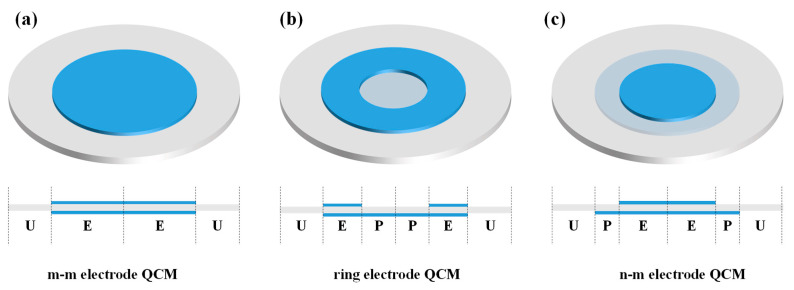
Schematic diagram of the structure of the (**a**) m-m electrode QCM, (**b**) ring electrode QCM, and (**c**) n-m electrode QCM. E, P, and U represent the electroded region, partially electroded region, and unelectroded region, respectively.

In general, a QCM humidity sensor consists of a quartz crystal resonator (QCR) and a humidity-sensing material deposited on its surface, as the bare QCR lacks selectivity for the target detection [12]. It is worth noting that the commonly used QCM humidity transducer has a symmetric electrode structure. The symmetrical electrode QCM, also called m-m electrode QCM, has circular electrodes of the same size on the upper and lower sides, see Figure 1a. Therefore, the frequency variation of the symmetric electrode QCM humidity sensor is mainly induced by the mass and viscosity effects of the humidity-sensing material when it adsorbs or desorbs water molecules, while the frequency shifts due to the change of its electrical parameters are almost neglected [13]. The frequency variation of the symmetrical electrode QCM humidity sensor is [14]:

(1) $$\Delta f=-\frac{2f_{0}^{2}\Delta m}{A{\left(\rho_{q}\mu_{q}\right)}^{1/2}}-{\left(\frac{\rho_{L}\eta_{L}}{2\rho_{q}\mu_{q}}\right)}^{1/2}f_{0}^{3/2}$$

where the negative sign means that the operating frequency of the QCM decreases. $f_{0}$, Δm, and A are the resonant frequency, mass change of the humidity-sensitive material, and effective sensing area, respectively, $\mu_{q}$ and $\rho_{q}$ are the shear modulus and density of the quartz crystal, respectively, and $\mu_{L}$ and $\rho_{L}$ are the viscosity and density of the humidity-sensitive layer, respectively.

Asymmetric electrode QCM sensors are also sensitive to the changes in electrical properties, such as conductivity and relative permittivity, because the upper surface within its partially electroded region is not shielded by the metal electrode, resulting in an enhanced fringing field effect [13,15,16,17]. Both the ring electrode QCM and the n-m electrode QCM are typical asymmetric electrode QCMs, see Figure 1b,c. The upper surface of the ring electrode QCM has a ring electrode, while the lower surface has a circular electrode. The n-m electrode QCM has circular electrodes of different diameters on both the upper and lower surfaces. Generally, the electrodes with smaller and larger diameters are referred to as n and m electrodes, respectively [18]. For n-m and m-m electrode QCMs, the diameters of the upper and lower electrodes are often used to directly represent the QCM, e.g., 3-5 QCM means that the diameters of its top and bottom circular electrode are 3 and 5 mm, respectively. 5-5 QCM means that the diameters of its top and bottom circular electrode are both 5 mm.

For QCM humidity sensors, the electrical parameters of the humidity-sensitive materials also change when adsorbing or desorbing water molecules, and this change affects the resonance state of the asymmetric electrode QCM humidity sensors, which in turn enhances their sensitivity. In 2021, Chen et al. proposed an asymmetric QCM humidity sensor with a ring electrode configuration [19]. Using lignin as the sensitive material, the humidity-sensing experimental results showed that the sensitivity of the ringed electrode QCM humidity sensor was more than twice that of the QCM humidity sensor with a symmetrical electrode configuration. The sensitivity was as high as 61 Hz/%RH with a fast response/recovery time of 28/5 s in the humidity range of 11.3% RH to 97.3% RH. Additionally, then, they analyzed the electric field and potential distribution of the ring and symmetric electrode structure using COMSOL Multiphysics software, and the simulation results showed the fringing field effect of the ring electrode QCM is significantly stronger than that of the symmetric electrode QCM. Finally, they used the Advanced Design System (ADS) software to illustrate that the change in the electrical parameters of the sensitive material brings additional frequency changes for the ringed electrode QCM humidity sensor through the equivalent circuit. In addition, another asymmetric n-m electrode QCM humidity sensor was constructed by Yao et al. [13]. They found that the asymmetric electrode configuration greatly enhanced the sensitivity of the QCM humidity sensor through experiments and equivalent circuit analysis. The maximum sensitivity was up to 119.7 Hz/%RH with a response/recovery time of 60/15 s in the range of 11.3% RH to 97.3% RH. Unfortunately, Yao et al. did not discuss the potential mechanism of the asymmetric n-m electrode QCM humidity sensor in detail.

Chitin is a rich natural polymeric polysaccharide, which is the main structural component of crustacean shells. Chitin nanofibers treated by hydrolysis and ultrasonication have excellent properties such as low bulk density, large surface area, good chemical reactivity, low toxicity, biodegradability, biocompatibility, antioxidant activity, antibacterial properties, and excellent mechanical properties, which is well suited as moisture-sensitive materials [20,21,22,23,24]. In this paper, the humidity-sensitive characteristics of QCM sensors with the asymmetric n-m electrode and symmetric electrode were compared using chitin nanofibers as a moisture-sensitive material combined with impedance analysis and oscillating circuit method. Then, the possible mechanism of the ChNFs-integrated n-m electrode QCM humidity sensor is analyzed in detail. The mass sensitivities of the n-m electrode and symmetric electrode QCMs were then calculated and compared by solving for the particle displacement amplitude. The electric potential and electric field distributions of the asymmetric and symmetric n-m electrode structures were analyzed with the finite element analysis. Finally, breath testing was used as an example to show its potential application.

## 2. Materials and Methods

**Figure 2:**
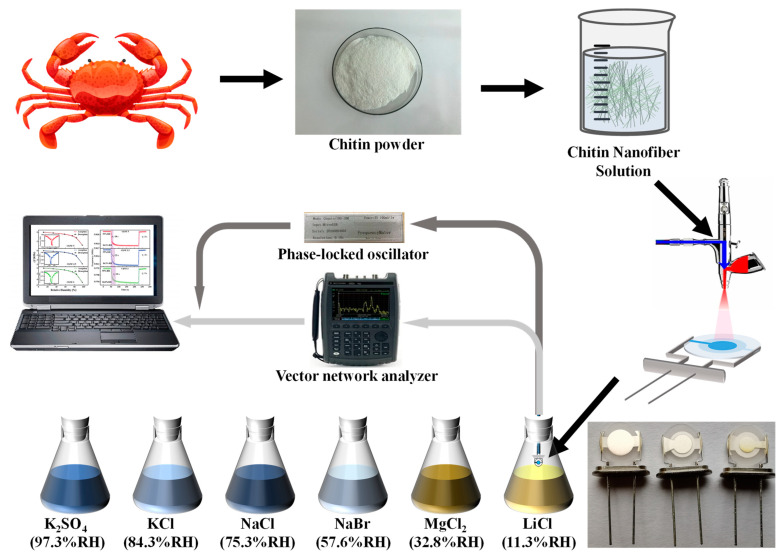
Schematic diagram of the experimental setup.

The experimental setup is schematically shown in Figure 2. Crab shell chitin powder (2.2 g) was mixed with TEMPO (0.04 g), and NaBr (0.20 g) in deionized water (197.8 g). NaClO solution (30.0 g, 10–13% aqueous solution) was added and stirred continuously for 24 h. The pH was maintained between 10.0 and 10.3 by adding NaOH solution (0.5 mol/L). Ten milliliters of ethanol were added to terminate the reaction and then stirred for 20 min. After five rounds of washing with deionized water and centrifugation to remove the completed product from the reaction mixture, the supernatant—which included the aqueous suspension containing the chitin nanofibers—was collected and freeze-dried to obtain chitin nanofiber powder. The aqueous solution of ChNF was stained with phosphotungstic acid and then dropped onto a copper grid and dried at ambient temperature. Transmission electron microscopy (TEM, Philips CM10, FEI Company, Eindhoven, The Netherlands) was used to observe its microscopic morphology. The Fourier transform infrared spectroscopy (FTIR) and water contact angle of ChNF were characterized using a Nicolet iS10 (Thermo Fisher Scientific Inc., Madison, WI, USA) and contact angle goniometer (DSA30, Kruss, Hamburg, Germany) were characterized, respectively.

The AT-cut, 6 MHz QCMs with different electrode structures were fabricated at Wuhan Hitrusty Electronics Co., Ltd. (Wuhan, China). The diameter of the quartz wafer was 8 mm. The symmetric and asymmetric electrode structures were formed by depositing circular electrodes of different diameters on both sides of the quartz wafer using a thermal evaporation method. The silver electrode on one side has a diameter of 5 mm, and for comparison purposes, the silver electrodes on the other side have diameters of 3, 3.5, and 5 mm, respectively, and were also labeled 3-5 QCM, 3.5-5 QCM, and 5-5 QCM, respectively. The equivalent circuit parameters of QCM with symmetric and asymmetric electrode structures were tested before depositing the ChNF films, as shown in Table 1. A 4 mm circular of ChNFs was deposited on the surface of the QCM sensor with a small diameter using the spray method. The spray time was chosen to be 40 s to ensure that the same amount of ChNFs was deposited for all sensors. After drying, QCM humidity sensors were obtained and labeled as ChNF-3, ChNF-3.5, and ChNF-5, respectively.

The experimental equipment mainly consisted of humidity generation devices, a phase-locked oscillator (PLO, Chengdu Leopold Technology Co., Ltd., Chengdu, China), a vector network analyzer (VNA, keysight N9913A, Keysight Technologies, Inc., Santa Rosa, CA, USA), and a personal computer (PC). The PLO and VNA were each linked to the PC for recording and evaluating the humidity sensing performance and resonance state of the ChNFs integrated QCM sensors. The fixed humidity levels were produced by saturated K_2_SO_4_ solution (97.3% RH), KCl solution (84.3% RH), NaCl solution (75.3% RH), NaBr solution (57.6% RH), MgCl_2_ solution (32.8% RH), and LiCl solution (11.3% RH) at 25 °C, respectively.

## 3. Results and Discussion

**Figure 3:**
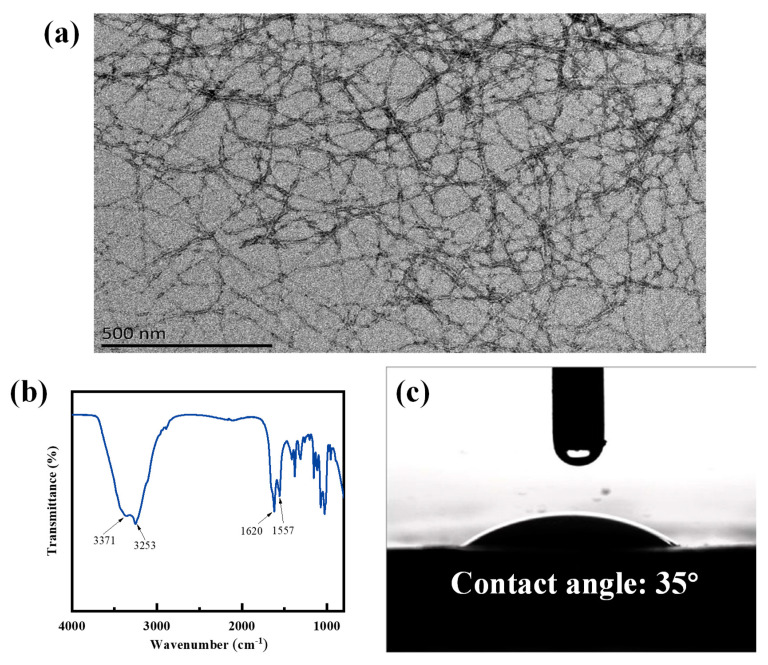
(**a**) TEM, (**b**) FTIR, and (**c**) water contact angle of the prepared ChNFs.

The TEM image of the chitin nanofiber is displayed in Figure 3a. ChNF exhibits a hair-like cross-network structure with a diameter of about 20–30 nm, so it has a very high specific surface area, which creates more active sites to adsorb more water molecules. As shown in Figure 3b, 1620 cm^−1^ and 1557 cm^−1^ in the FTIR spectrum are two typical characteristic peaks of chitin, mainly attributed to the C=O stretching of amide I, and the combination of N-H bending vibrations with C-N and N-H vibrations in amide II, respectively [22]. Overall, 3373 cm^−1^ and 3253 cm^−1^ are attributed to the O-H and N-H stretching vibrations, respectively, which are typical hydrophilic groups [22,25]. Generally, when the water contact angle of the material is less than 90°, it is considered to have good hydrophilicity [26]. Figure 3c shows that the water contact angle of ChNF is about 35°, indicating its excellent hydrophilicity, implying that it is well suited as a moisture-sensitive material.

**Figure 4:**
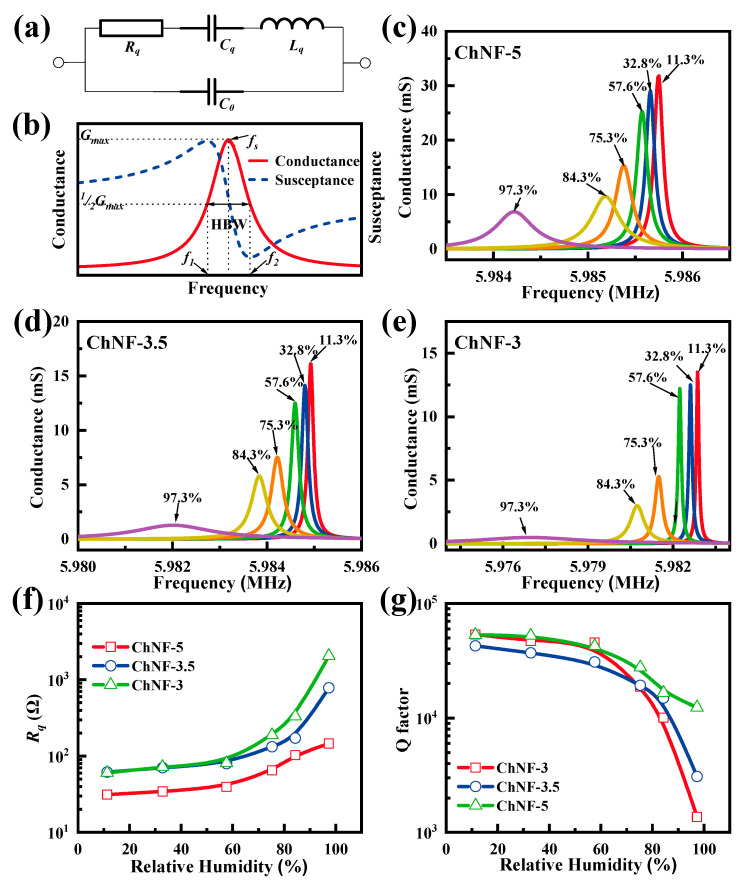
(**a**) Schematic of the Butterworth–Van Dyck (BVD) equivalent circuit, and (**b**) the typical conductance and susceptance of the QCM sensor. The (**c**–**e**) conductances, (**f**) dynamic resistances, and (**g**) Q-factors of the ChNF-5, ChNF-3.5, and ChNF-3.

The Butterworth–Van Dyke (BVD) model illustrated in Figure 4a is often employed to characterize the electro-acoustic impedance behavior of the QCM sensors [27,28,29].$\;C_{0}$,$\;C_{q}$, $L_{q}$, and $R_{q}\;$ are related to the additional capacitance between the two electrodes, mechanical flexibility, initial mass, and energy dissipation of the QCM sensor, respectively. Figure 4b shows a typical electroacoustic spectrum of QCMs, i.e., conductance and susceptance. The frequency corresponding to the peak of the conductance curve is considered as the operating frequency (f) of the QCM sensor. Figure 4c–e plot the conductances of all ChNF-integrated QCM humidity sensors in different humidity environments. The Q value, which can be estimated by dividing the operating frequency by the half bandwidth of the conductivity curve (HBW), is closely related to the stability of QCM sensors. It can be found that the peak frequency (f) of the conductance curve of each sensor decreases and the HBW widens as the humidity increases. The equivalent circuit parameters and Q values of the ChNF-integrated QCM sensors were obtained from the conductance and susceptance [27], as shown in Table 2. As shown in Figure 4f, the equivalent resistance ($R_{q}$) of all ChNF-integrated QCM humidity sensors increases with increasing humidity. The $R_{q}$ of the n-m electrode QCM sensors are significantly larger than m-m electrode QCM sensors, and the smaller the electrode area, the larger the $R_{q}$ in a high humidity environment, which means that the ChNF-3 requires higher energy. The following equation is often employed to calculate the Q value of the QCM sensors:

(2) $$Q=\frac{\omega L_{q}}{R_{q}}=\frac{2\pi fL_{q}}{R_{q}}$$

As shown in Figure 4g, the Q values of all sensors decrease as the humidity increases. The Q values of the n-m electrode QCM are significantly lower than that of the symmetric electrode QCM, and the smaller the diameter of the n electrode, the lower the Q values of the n-m electrode QCM.

**Figure 5:**
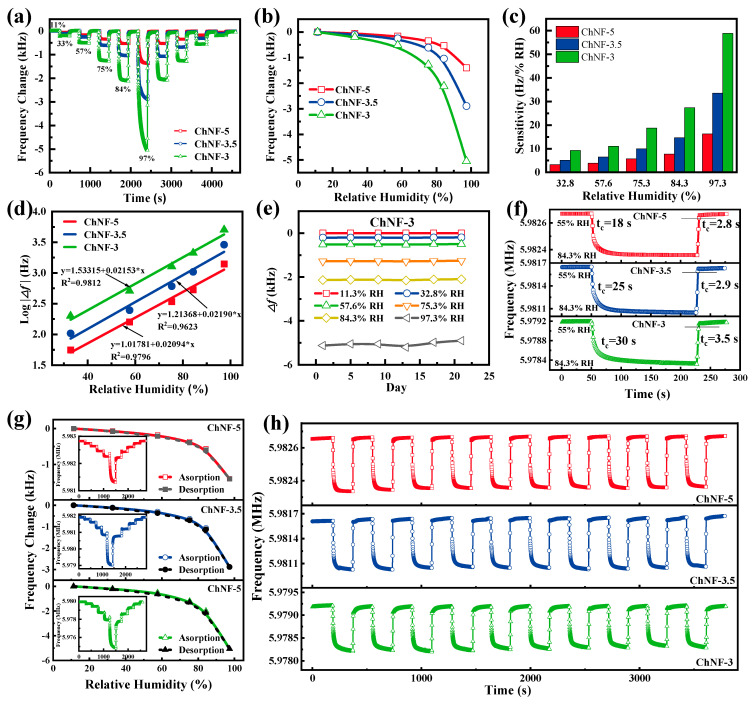
The humidity-sensing properties of ChNF-integrated QCM sensors: (**a**) dynamic response, (**b**) static frequency response (**c**) sensitivity variation, (**d**) fitted curve of log|Δf| vs. RH, (**e**) long-term stability, (**f**) humidity hysteresis, (**g**) response/recovery times, and (**h**) repeatability.

The dynamic responses of ChNF-3, ChNF-3.5, and ChNF-5 are in Figure 5a. All ChNF integrated QCM sensors are well responsive to the variation of relative humidity. The good consistency of increasing and decreasing frequency changes during the dynamic changes of relative humidity indicates excellent invertibility and stability. The static frequency responses of ChNF-5, ChNF-3.5 and ChNF-3 from 11.3% RH to 97.3% RH are given in Figure 5b. The frequency change of ChNF-3 is bigger than that of ChNF-3.5 and ChNF-5 at each humidity point, indicating that ChNF-3 has the highest humidity sensitivity and ChNF-5 has the lowest sensitivity. This result indicates that the asymmetric electrode structure effectively enhances the sensitivity of the QCM humidity sensor. Additionally, the smaller the area of the n electrode, the higher the sensitivity of the n-m electrode QCM. However, it is worth noting that a smaller electrode area also results in lower Q values for the n-m electrode QCM humidity sensor. Therefore, it is necessary to select an appropriate electrode diameter to balance the sensitivity and stability of the QCM humidity sensor.

The sensitivity for frequency-response humidity sensors is usually defined as the ratio of the frequency shift to the change in humidity levels. As shown in Figure 5c, the maximum frequency changes of ChNF-5, ChNF-3.5, and ChNF-3 at 97.3% RH were 1400, 2880, and 5060 Hz, respectively, and their sensitivity can be calculated as 16.28, 33.49, and 58.84 Hz/%RH, respectively. As shown in Figure 5d, the frequency responses of all ChNF-integrated QCM humidity sensors show a logarithmic relationship with relative humidity with correlation coefficients (R^2^) of 0.9796, 0.9623, and 0.9812, respectively, like most published humidity sensors [30,31,32,33,34]. Figure 5e exhibits a very small frequency fluctuation of the ChNF-3 over 21 days, which implies its superb long-term stability.

The maximum frequency differences of ChNF-5, ChNF-3.5, and ChNF-3 are 29, 59, and 125 Hz, thus the humidity hysteresis of the sensors can be calculated as 2.0%, 2.1%, and 2.5 % RH at the humidity environment of 57.6% RH, respectively (Figure 5e). As shown in Figure 5g, the response/recovery times of the ChNF-5, ChNF-3.5, and ChNF-3 humidity sensors were 18/2.8, 25/2.9, and 30/3.5 s, respectively, over the range from ambient humidity (55% RH) to 84.3% RH. However, it is worth noting that, although the asymmetric n-m electrode structure enhances the frequency response, the response time is also prolonged. The repeatabilities of the chitin nanofiber-integrated QCM sensors are shown in Figure 5h, and the frequency changes of ChNF-5, ChNF-3.5, and ChNF-3 show a negligible change in 10 cycles between ambient humidity (55% RH) and a humidity environment of 84.3% RH, which demonstrates the good repeatability of the chitin nanofiber-coated QCM humidity sensors. Table 3 and Table 4 show the performance comparison of the ChNF-3 with some published humidity sensors. The ChNF integrated n-m electrode QCM humidity sensor is extremely competitive in terms of sensitivity, humidity hysteresis, and response/recovery times [11,19,35,36,37,38,39,40,41,42,43,44].

## 4. Analysis of Sensitivity Enhancement Mechanisms

**Figure 6:**
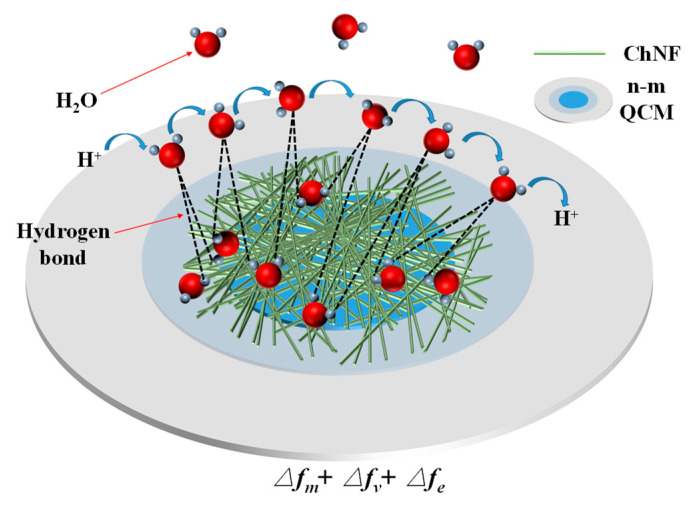
Schematic diagram of the humidity-sensitive mechanism of ChNFs-integrated n-m electode QCM sensors.

Figure 6 shows the schematic diagram of the possible moisture-sensing mechanism of the ChNFs integrated n-m electrode QCM humidity sensors. The FTIR spectra of chitin nanofibers showed that their surfaces are rich in hydrophilic groups such as amino and hydroxyl groups, so H_2_O molecules can be adsorbed on hydrophilic groups through hydrogen bonds. ChNF’s mass and viscosity will change as a result of the adsorption or desorption of H_2_O molecules, altering the Q value and operating frequency of the QCM. However, the electrical parameters of the ChNF film also change, thus affecting the fringing field distribution, and thereby changing the resonant state of the QCM.

Monolayer water molecules are adsorbed via chemical bonding on the surface of ChNF at low relative humidity. Additionally, the frequency and Q value of QCM decrease insignificantly at this stage. The H^+^ ions in the hydrophobic functional groups of ChNF combine with a tiny number of H_2_O molecules to create H_3_O^+^. As the humidity increases, ChNF adsorbs more water molecules, resulting in the formation of multilayer water molecules. At this stage, the sensitive film’s mass and viscosity start to rise even more, which lowers the resonance frequency and Q value of the QCM humidity sensors. Additionally, the electric field will cause the water molecules to become polarized, creating a significant amount of H_3_O^+^ ions [55]. At the high humidity stage, the mass and viscosity of ChNF increase as an exponential function due to the continuous superposition of liquid water molecule layers on its surface, which also well explains the logarithmic frequency response of the QCM humidity sensors. At this point, the hydroxy-functional groups of ChNF undergo hydrolysis reactions in these aqueous layers, leading to a large increase in the concentration of H^+^, which also greatly enhances the ionic conduction of ChNF films.

Since the asymmetric n-m electrode QCM humidity sensor has no metal electrode shading on the upper surface of the partially electroded area enhances its fringing field effect. As a result, frequency shifts associated with changes in the electrical characteristics of moisture-sensitive materials are created in addition to the frequency variations caused by mass and viscosity. Thus, the frequency variation of the asymmetric n-m electrode QCM humidity sensor contains three components, i.e., $\Delta f_{m}+\Delta f_{v}+\Delta f_{e}$. $\Delta f_{m}$, $\Delta f_{v}$, and $\Delta f_{e}$, are caused by changes in mass, viscosity, and electrical parameters, respectively, of the humidity-sensitive film during the adsorption or desorption of water molecules. Among them,$\;\Delta f_{e}$ depends mainly on the electrical parameters of the sensitive film and the fringing field effect of the QCM. The reasons why ChNF-3 has the highest humidity sensitivity will be discussed in detail below. Additionally, $\Delta f_{m}$ and $\Delta f_{v}$ are mainly determined by the QCM mass sensitivity.

**Figure 7:**
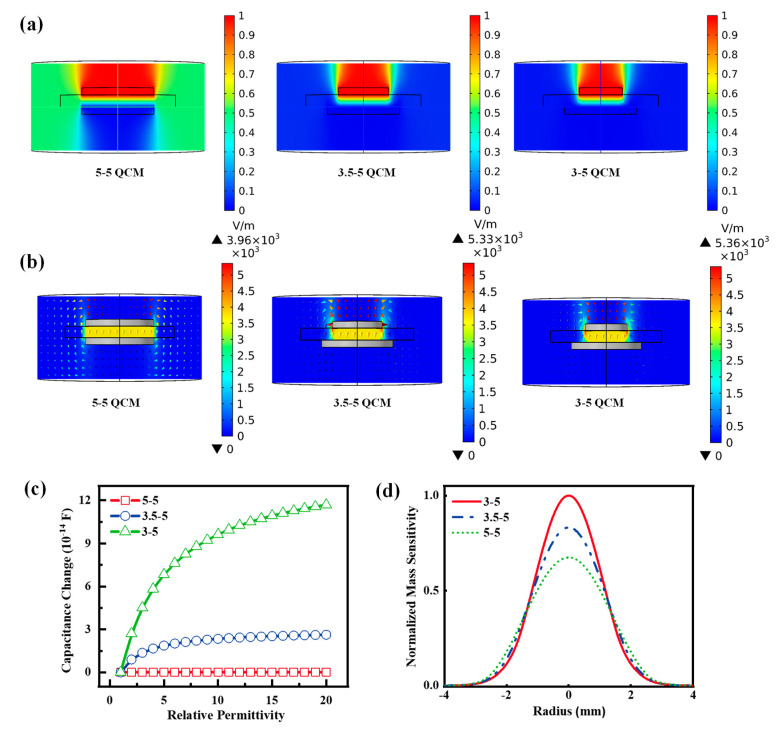
(**a**,**b**) The distribution of the potential and electric field of 5-5 QCM, 3.5-5 QCM, and 3-5 QCM. (**c**) The normalized mass sensitivity of the QCM with symmetric and asymmetric electrodes. (**d**) Simulation results of capacitance change VS relative permittivity for the 5-5, 3.5-5, and 5-5.

At first, for the asymmetric n-m electrode configuration, the partially electroded region is not shielded by the metal electrode because of the different electrode diameters at the top and bottom, which enhances its fringing field effect. The fringing field distributions of symmetric and asymmetric n-m electrode configurations were simulated using the finite element analysis method, and their potential and electric field distributions are given in Figure 7a,b. The fringing field effect of 3-5 QCM in the radius range of 1.5–2 mm is significantly larger than that of 3.5-5 QCM, while the 5-5 QCM is the smallest. The adsorption or desorption of water molecules at different humidity levels leads to changes in the relative permittivity of the sensitive material.

For simplicity, 4 mm diameter, 500 nm-thick cylinders were placed in the center of QCMs to simulate the moisture-sensitive layer. Figure 7c shows the change capacitance to 5-5 QCM, 3.5-5 QCM, and 3-5 QCM as the relative permittivity of the moisture-sensing layer varied from 1 to 20. The capacitance change for the asymmetric n-m electrode structure is larger than that of the symmetric electrode structure because of the enhanced fringing field effect. Additionally, the smaller the diameter of the upper electrode, the larger the capacitance of the asymmetric n-m electrode configuration. According to the equivalent circuit analysis of the asymmetric electrode QCM by Yao et al., the capacitance will also cause the frequency change of the QCM [13].

In another aspect, the mass sensitivities of QCM determining $\Delta f_{m}$ and $\Delta f_{v}$ were calculated and compared. The mass sensitivity, $S_{f}\left(r,\theta \right)$, of QCM can be determined by the following equation [18,56]:

(3) $$S_{f}\left(r\right)=\frac{\left|\tilde{u}\left(r\right)\right|}{2\pi \int_{0}^{\infty }r|\tilde{u}\left(r\right){|}^{2}dr}C_{f}$$

where $\tilde{u}\left(r\right)$ and $C_{f}$ are the particle displacement amplitude and Sauerbrey’s mass sensitivity constant, respectively [57]. The $\tilde{u}\left(r\right)$ can be solved by the following Bessel equation.

(4) $$r^{2}\frac{\partial^{2}\tilde{u}\left(r\right)}{\partial r^{2}}+\frac{\partial \tilde{u}\left(r\right)}{\partial r}+\frac{k_{i}^{2}r^{2}}{N}\tilde{u}\left(r\right)=0$$

*N* is obtained from the material constant of the piezoelectric quartz crystal. $k_{i}^{2}=\left(\omega^{2}-\omega_{i}^{2}\right)/c^{2}$, where *i* = *E* (fully electroded region), *P* (partially electroded region), and *U* (non-electroded region); c is the velocity of the wave through the quartz crystal as a function of the material parameters: $c=\sqrt{c_{66}/\rho_{q}}$. $\omega_{i}$ is the cutoff frequency in each region [58].

The mass sensitivity distributions of the 3-5 QCM, 3.5-5 QCM, and 5-5 QCM were calculated through the parameters of the AT-cut, 6 MHz QCM, as shown in Figure 7d. The 3-5 QCM has the highest mass sensitivity, followed by the 3.5-5 QCM, and the 5-5 QCM has the lowest. This result indicates that the asymmetric n-m electrode configuration enhances the mass sensitivity. Additionally, the smaller the upper electrode diameter, the larger the mass sensitivity of the asymmetric n-m electrode QCM. In brief, 3-5 QCM has the highest mass sensitivity and electrical sensitivity, so ChNF-3 has the highest humidity sensitivity.

## 5. Sensor Applications

**Figure 8:**
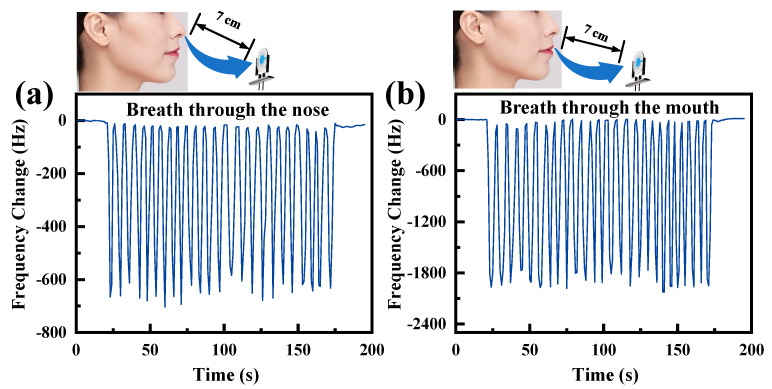
Human breathing monitoring with ChNF-integrated QCM sensors: (**a**) mouth breathing, (**b**) nose breathing.

Breath detection is beneficial in monitoring human signs and early medical diagnosis. In this paper, we investigate the potential application of the ChNF integrated QCM humidity sensor for respiratory detection. The sensor was placed 7 cm away from the nose and mouth for testing, and the results are shown in Figure 8a,b. The frequency shifts caused by nose breathing and mouth breathing were about 650 Hz and 2000 Hz, respectively. This can be explained by the fact that the humidity change of nose breathing is smaller than that of mouth breathing [41]. Due to the different humidity of the exhaled gas, the patterns of mouth breathing and nose breathing can be distinguished. Therefore, the ChNF integrated QCM humidity sensor has promising applications for human respiration monitoring.

## 6. Conclusions

In summary, three QCM humidity sensors were fabricated by spraying chitin nanofibers on the surface of AT-cut, 6 MHz QCM transducers with different upper electrode diameters. The QCM humidity sensor having a smaller electrode area exhibited high sensitivity of 58.84 Hz/%RH, competitive response/recovery time of 30/3.5 s, and low humidity hysteresis of 2.5% RH. However, it is necessary to choose a suitable electrode diameter to balance the stability and sensitivity because the impedance analysis result showed that the reduction of the electrode diameter leads to a sharp decrease in the Q value of the QCM. Next, the possible humidity sensing mechanism of the ChNF-integrated asymmetric n-m electrode QCM humidity sensor was discussed. Since the asymmetric n-m electrode QCM humidity sensor has no metal electrode shading on the upper surface of the partially electroded area enhances its fringing field effect. The mass sensitivities of the 3-5 QCM, 3.5-5 QCM, and 5-5 QCM were calculated from the mass displacement amplitude, and it was found that the 3-5 QCM has the highest mass sensitivity. Therefore, frequency shifts associated with changes in the electrical characteristics of moisture-sensitive materials are created in addition to the frequency variations caused by mass and viscosity. Finally, we demonstrated the potential application of the ChNF integrated QCM humidity sensor through breath detection experiments. This work not only demonstrates that chitin nanofibers are an excellent potential material for moisture detection, but also provides a new perspective for designing high-performance QCM humidity sensors.

## Figures and Tables

**Table 1:** Equivalent circuit parameters of the bare QCM in the experiment.

	$R_{q}$ (Ω)	$L_{q}$ (mH)	$C_{q}$ (fF)	$C_{0}$ (pF)	Q
ChNF-5	29.1729	4.7402	14.9185	4.2481	47,084
ChNF-3.5	54.1703	7.1384	9.9066	3.2460	49,529
ChNF-3	61.9854	8.5007	8.3190	2.9921	51,544

**Table 2:** The equivalent circuit parameters of ChNF-5, ChNF-3.5, and ChNF-3 at each humidity level.

		$R_{q}$ (Ω)	$L_{q}$ (mH)	$C_{q}$ (fF)	$C_{0}$ (pF)	Q
ChNF-5	11.30%	31.29	44.38	15.95	4.34	53,283
	32.80%	34.42	48.15	14.7	4.01	52,553
	57.60%	39.5	44.79	15.8	4.32	42,603
	75.30%	65.15	48.07	14.72	4.03	27,720
	84.30%	103.46	45.65	15.5	4.26	16,576
	97.30%	146.32	48.21	14.69	4.04	12,378
ChNF-3.5	11.30%	61.81	70.21	10.08	3	42,669
	32.80%	70.76	69.8	10.14	3.02	37,058
	57.60%	80	65.67	10.78	3.2	30,835
	75.30%	133.08	68.25	10.37	3.08	19,263
	84.30%	172.94	68.63	10.32	3.06	14,905
	97.30%	782.86	64.26	11.03	3.21	3082
ChNF-3	11.30%	60.34	86.2	8.22	2.88	53,649
	32.80%	72.45	90.9	7.79	2.74	47,117
	57.60%	81.26	98.56	7.19	2.52	45,544
	75.30%	189.27	94.42	7.51	2.6	18,731
	84.30%	334.81	89.92	7.88	2.68	10,082
	97.30%	2061.53	75	9.46	2.98	1365

**Table 3:** Comparison of humidity sensing performance between ChNF-integrated n-m electrode QCM sensors and other QCM humidity sensors.

Materials	Sensing Range (RH)	Sensitivity (ppm/%RH)	Response/ Recovery Time (s)	Humidity Hysteresis (%RH)	Reference
Lignin	11.3–97.3%	6.1	29/5	6.2	[19]
CNCs	11.3–97.3%	12.0	60/15	7.3	[13]
S-Ti3C2	11.3−97.3%	1.28	6/2	1.16	[35]
GO	11.3−97.3%	2.21	45/21	Not given	[36]
SnO2	11.3−97.3%	2.9	10/3	Not given	[37]
NCNCs	11.3−84.3 %	1.3	18/10	1.6	[38]
green microspheres	11.3−97.3%	3.0	48/65	0.08	[39]
Sb/WO3	0–85%RH	3.6	10/1.6	Not given	[11]
multi-pore PDA	11−97.3%	0.5	12/39	3.66	[40]
PDA/GO	0–97.3%	12.5	18/2	2.1	[41]
PDA@CNCs/GO	11.3–97.3%	5.5	37/5	4.3	[42]
CNT	5–97%	0.5	60/70	Not given	[43]
MWCNTs-CS	11–95%	4.7	75/34	0.8	[44]
Chitin nanofiber	11.3–97.3%	9.8	30/3.5	2.5	This work

**Table 4:** Comparison of humidity sensing performance between ChNF-integrated n-m electrode QCM sensors and other types of humidity sensors.

Materials	Sensing Principle	Sensing Range (RH)	Sensitivity (Operating Frequency)	Response/ Recovery Time (s)	Humidity Hysteresis (%RH)	Reference
PVA/GF	Capacitance	40–90%	29 nF/%RH (10 kHz)	2/3.2	Not given	[45]
Potato peel	Impedance	10–90%	70 kΩ/% RH (1 kHz)	8/12	2.1	[46]
ZnO/GrF	Resistance	15–86%	7.7 µA/%RH (Not give)	0.4/4	Not given	[47]
egg white	Impedance	10–85%	50 kΩ/%RH (1 kHz)	1.2/1.7	Not given	[48]
P(VDF-TrFE)/GF	Capacitance	8–98%	0.056 pF/%RH (10 kHz)	0.8/2.5	>20	[49]
GO	CMUT	22.5–43.2%	241.67 ppm/%RH (10 MHz)	10/4	Not given	[50]
PVA	SAW	0–98.8%	7.35 kHz/%RH (433 MHz)	35/46	0.004	[51]
PI	FBAR	15–85%	67.3 KHz/%RH (1055 MHz)	17/26	1.77	[52]
GO	Cantilever	10–90	84 Hz/%RH (2 MHz)	17/12	<3	[53]
MoO3	SIW	10–90	2.062 MHz/%RH (9.1 GHz)	3/2	0.25	[54]
Chitin nanofiber	QCM	11.3–97.3%	58.84 Hz/%RH (6 MHz)	30/3.5	2.5	This work

## Data Availability

Not applicable.
